# Mutations Conferring Increased Sensitivity to Tripartite Motif 22 Restriction Accumulated Progressively in the Nucleoprotein of Seasonal Influenza A (H1N1) Viruses between 1918 and 2009

**DOI:** 10.1128/mSphere.00110-18

**Published:** 2018-04-04

**Authors:** Isabel Pagani, Andrea Di Pietro, Alexandra Oteiza, Michela Ghitti, Nadir Mechti, Nadia Naffakh, Elisa Vicenzi

**Affiliations:** a San Raffaele Scientific Institute, Milan, Italy; b CNRS UMR 5235, DIMNP, University of Montpellier, Montpellier, France; c Institut Pasteur, Unité de Génétique Moléculaire des Virus à ARN, Département de Virologie, Paris, France; d CNRS, UMR3569, Paris, France; e Université Paris Diderot, Sorbonne Paris Cité, Paris, France; University Medical Center Freiburg

**Keywords:** influenza A virus, TRIM22, evolution, nucleoprotein, restriction

## Abstract

Influenza A viruses (IAVs) can cause zoonotic infections with pandemic potential when most of the human population is immunologically naive. After a pandemic, IAVs evolve to become seasonal in the human host by acquiring adaptive mutations. We have previously reported that the interferon (IFN)-inducible tripartite motif 22 (TRIM22) protein restricts the replication of seasonal IAVs by direct interaction with the viral nucleoprotein (NP), leading to its polyubiquitination and proteasomal degradation. Here we show that, in contrast to seasonal H1N1 IAVs, the 2009 pandemic H1N1 strain as well as H1N1 strains from the 1930s are resistant to TRIM22 restriction. We demonstrate that arginine-to-lysine substitutions conferring an increased sensitivity to TRIM22-dependent ubiquitination accumulated progressively in the NP of seasonal influenza A (H1N1) viruses between 1918 and 2009. Our findings suggest that during long-term circulation and evolution of IAVs in humans, adaptive mutations are favored at the expense of an increased sensitivity to some components of the innate immune response.

**IMPORTANCE** We have uncovered that long-term circulation of seasonal influenza A viruses (IAV) in the human population resulted in the progressive acquisition of increased sensitivity to a component of the innate immune response: the type I interferon-inducible TRIM22 protein, which acts as a restriction factor by inducing the polyubiquitination of the IAV nucleoprotein (NP). We show that four arginine residues present in the NP of the 1918 H1N1 pandemic strain and early postpandemic strains were progressively substituted for by lysines between 1918 and 2009, rendering NP more susceptible to TRIM22-mediated ubiquitination. Our observations suggest that during long-term evolution of IAVs in humans, variants endowed with increased susceptibility to TRIM22 restriction emerge, highlighting the complexity of selection pressures acting on the NP.

## Abstract (precis)

We have uncovered that long-term circulation of seasonal influenza A viruses (IAV) in the human population resulted in the progressive acquisition of increased sensitivity to a component of the innate immune response: the type I interferon-inducible TRIM22 protein, which acts as a restriction factor by inducing the polyubiquitination of the IAV nucleoprotein (NP). We show that four arginine residues present in the NP of the 1918 H1N1 pandemic strain and early postpandemic strains were progressively substituted for by lysines between 1918 and 2009, rendering NP more susceptible to TRIM22-mediated ubiquitination. Our observations suggest that during long-term evolution of IAVs in humans, variants endowed with increased susceptibility to TRIM22 restriction emerge, highlighting the complexity of selection pressures acting on the NP.

## INTRODUCTION

Influenza is an infectious disease caused by RNA viruses of the *Orthomyxoviridae* family. Influenza A viruses (IAVs) originate from wild aquatic birds and circulate among several other species, including chickens, pigs, and humans. The animal reservoir is a never-ending source of viruses that spill over to humans, causing mainly sporadic, self-limiting infections with minimal evidence of human-to-human transmission (1). Episodically, however, zoonotic IAVs evolve into pandemic strains which spread globally, causing significant death and illness. In general, an influenza pandemic occurs when most of the human population has no immunological memory against the emerging IAV strain (2); however, preexisting and cross-reactive immunity can prevent or attenuate the infection of the newly introduced virus in some sections of the population (3, 4). Pandemic viruses subsequently evolve into seasonal, human-adapted viruses, and “herd” immunity becomes established (5).

To efficiently replicate in the new host, pandemic IAVs must counteract a number of host restriction factors (RFs) that are mostly inducible by type I interferons (IFNs) produced during the early phase of the infection (6, 7). Among the most potent and best characterized type I IFN-stimulated genes (ISGs), the MxA gene encodes a dynamin-like large GTPase that recognizes the viral ribonucleoprotein (vRNP) complex, thereby blocking its function (8). Interestingly, it has been shown that while avian IAVs are generally highly sensitive to MxA restriction, pandemic strains escape MxA restriction through adaptive mutations in the viral nucleoprotein (NP) (9, 10). Another ISG, coding for the 3′-to-5′ exoribonuclease ISG20, was shown to inhibit IAV replication by interacting with NP (11). These observations suggest that NP is a major target of IFN-induced RF.

Accordingly, we have shown previously that tripartite motif 22 (TRIM22), a member of the large family of TRIM proteins, restricts IAV infection by interacting with the NP of seasonal viruses, thereby promoting its ubiquitination and proteasomal degradation (12). Here, we report that the 2009 pandemic H1N1 strain, as well as two IAV strains isolated in 1933 and 1934 that derive from the 1918 pandemic virus, are resistant to TRIM22 restriction. Sequence alignment revealed the presence of four arginine residues in the NP of TRIM22-resistant viruses instead of four lysine residues present in TRIM22-susceptible ones. All of these lysines are solvent exposed in the available NP crystal structure and therefore can serve as ubiquitin acceptor sites through the E3 ubiquitin ligase activity of TRIM22. Overall, our study provides a novel molecular signature for IAVs that are long established in the human population, which is, unexpectedly, associated with increased susceptibility to TRIM22-mediated restriction.

## RESULTS

### Acquisition of TRIM22 restriction upon long-term circulation of IAV in humans.

**FIG 1:**
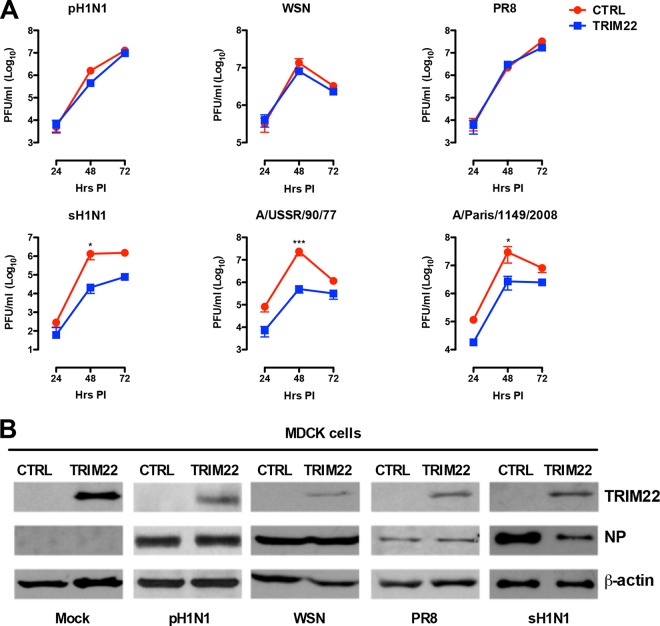
The pandemic 2009 H1N1 and H1N1 viruses from the 1930s are resistant to TRIM22 restriction. (A) Control and human TRIM22-transduced MDCK cells were infected with H1N1 strains at an MOI of 0.001. Viral titers were measured in supernatants harvested 24, 48, and 72 hpi by plaque assay, indicated as plaque-forming units (PFU). As a positive control for TRIM22 restriction activity, A/New Caledonia/20/99 (sH1N1) and two additional seasonal H1N1 strains, A/USSR/90/77 and A/Paris/1149/2008, were used to infect TRIM22-overexpressing MDCK cells. Viral titers are expressed as the means ± standard errors of the means (SEM) from three experiments performed in duplicate. *P* values were determined using two-way ANOVA (*, *P* < 0.05; ***, *P* < 0.001). (B) Total cell extracts from control and TRIM22-overexpressing MDCK cells infected with different H1N1 strains were analyzed by Western blotting, and viral NP expression levels were evaluated. TRIM22 expression was also examined, and β-actin was used as a normalizer. These results are representative of one out of three independent experiments.

TRIM22 was previously shown to inhibit the replication of seasonal IAV strains (12); in order to test whether it could also interfere with the replication of the 2009 pandemic H1N1 strain, MDCK cells stably overexpressing a TRIM22 protein fused to the HA tag or control MDCK cells were infected at low multiplicity of infection (MOI [0.001]) with A/Paris/7608/2009 virus (pH1N1). As controls, the two H1N1 viruses isolated in the 1930s, A/WSN/33 (WSN) and A/Puerto Rico/8/34 (PR8), and three more recent seasonal H1N1 viruses, A/New Caledonia/20/99 (sH1N1), A/USSR/90/77, and A/Paris/1149/2008, were tested in parallel. Culture supernatants were collected 24, 48, and 72 h postinfection (hpi), and the infectious titers were determined by plaque assay. Unexpectedly, the pH1N1, WSN, and PR8 viruses were insensitive to TRIM22 restriction (Fig. 1A), whereas TRIM22 overexpression significantly inhibited the replication of all more recent H1N1 viruses by ca. 1 log_10_ as we have originally reported (12).

As the TRIM22-mediated restriction of seasonal IAV is caused by TRIM22-dependent polyubiquitination and proteasomal degradation of NP (12), the levels of NP were determined in control and TRIM22-overexpressing MDCK cells 24 hpi with the different IAVs. As expected, the NP accumulation levels were lower in MDCK-TRIM22 cells infected with sH1N1 (A/New Caledonia/20/99) than control MDCK cells, whereas no such difference was observed in cultures infected with pH1N1, WSN, and PR8 strains (Fig. 1B).

These results indicate that the 2009 pandemic H1N1 and early derivatives of the 1918 pandemic H1N1 strains are insensitive to TRIM22 restriction, while IAVs that are long established in the human population have acquired susceptibility to this antiviral mechanism.

**FIG 2:**
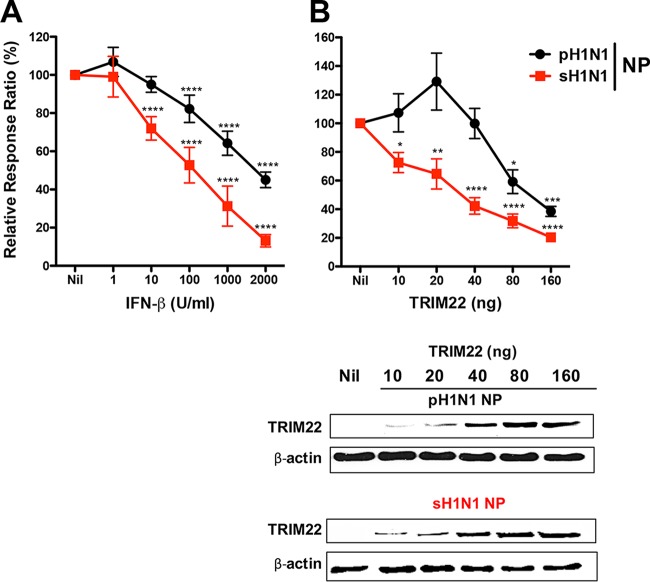
pH1N1 NP is more resistant to TRIM22-mediated inhibition than sH1N1 NP in a polymerase activity assay. Expression plasmids for the polymerase subunits PB2, PB1, and PA from the pH1N1 virus and NP expression plasmids from either the pH1N1 or sH1N1 strains were cotransfected with a plasmid carrying a pseudoviral genome, harboring a *Renilla* reporter, into HEK293T cells either without (Nil) or with increasing concentrations of TRIM22-expressing plasmid. The reporter gene activity was measured 24 h later. (A) Increasing amounts of exogenous beta interferon (IFN-β) were added to the polymerase activity assay. The sH1N1 NP-dependent polymerase activity was more sensitivity to the IFN-β stimulation than that of pH1N1 NP. Means ± standard deviations (SD) from 3 independent experiments in triplicates are reported. *P* values were determined using one-way ANOVA with Bonferroni’s multiple-comparison test of Nil versus each treatment (****, *P* < 0.0001). (B) Forty nanograms of TRIM22-expressing plasmid reduced the polymerase activity driven by the sH1N1 NP by 50% but not the polymerase activity driven by the pH1N1 NP. Means ± SEM from 3 independent experiments in triplicate are reported. *P* values were determined using one-way ANOVA with the Bonferroni’s multiple-comparison test of nil versus each treatment (*, *P* < 0.05; **, *P* < 0.01; ***, *P* < 0.001; ****, *P* < 0.0001). Western blot analysis was performed to determine the expression levels of TRIM22 and both pH1N1 and sH1N1 NPs.

We next evaluated TRIM22 restriction in a cell-based polymerase activity assay with either pH1N1 or sH1N1 NP. Expression plasmids for the pH1N1 polymerase subunits PB1, PB2, and PA were cotransfected into HEK293T cells together with an expression plasmid for either the pH1N1 or the sH1N1 NP and a plasmid driving the synthesis of a pseudoviral genomic RNA harboring a *Renilla* luciferase reporter gene. As the TRIM22 gene is one of the most strongly induced ISGs (13), we first tested whether IFN-β treatment affected the polymerase activity. As shown in Fig. 2A, at 10 U/ml of IFN-β the polymerase activity was inhibited in the presence of sH1N1 NP (squares), but not in the presence of pH1N1 NP (circles). At higher IFN-β concentrations, the polymerase activity was inhibited by both NPs, but to a lesser extent in the presence of the pH1N1 NP.

We then transfected increasing amounts (10 to 160 ng) of a TRIM22 expression plasmid together with the vRNP components, which resulted in increasing steady-state levels of TRIM22 as assessed by Western blotting (Fig. 2B, bottom panel). In the presence of sH1N1 NP (squares), the polymerase activity was inhibited by TRIM22 expression in a dose-dependent manner, whereas the pH1N1 NP rendered the system quite insensitive to TRIM22-mediated inhibition (Fig. 2B, upper panel).

These results strongly suggest that differences between the amino acid sequences of pH1N1 and sH1N1 NP determine the sensitivity to TRIM22-mediated restriction.

### Identification of four arginine-to-lysine mutations occurring during NP evolution.

**FIG 3:**
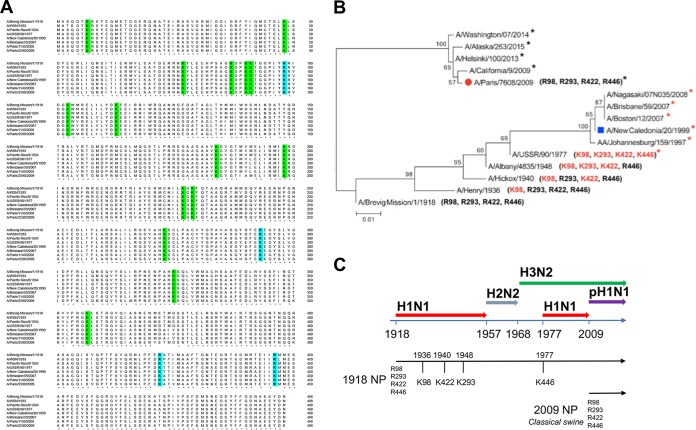
NP sequence alignment of human H1N1 strains and phylogenetic analysis of representative NP from pandemic and seasonal H1N1 strains. (A) The sequence comparison of NP from H1N1 strains (A/Brevig Mission/1918, A/WSN/33, A/PR/8/34, A/USSR/90/77, A/New Caledonia/20/99, A/Brisbane/59/2007, A/Paris/1149/2008 and A/Paris/2590/2009) shows 15 conserved lysine residues (in green). In A/New Caledonia/20/1999 and A/Brisbane/59/2007 isolates, arginine residues have been substituted for by lysines (in blue) at four specific sites (K98, K293, K422, and K446). Four arginine residues (R98, R293, R422, and R446) are present in the pandemic A/Paris/2590/2009 strain. (B) The maximum likelihood tree of 12 selected sequences shows two major branches that separate the H1N1 strains derived from the 1918 Brevig H1N1 strain from those derived from the swine 2009 pandemic H1N1 strain. Numbers at the nodes indicate bootstrap values obtained after 1,000 replications. A red asterisk indicates a viral isolate with four lysine residues, whereas a black asterisk indicates a viral isolate with four arginine residues. (C) Three major pandemics occurred during the 20th century, including the “Spanish” influenza pandemic (H1N1, 1918), the “Asian” pandemic (H2N2, 1957), and the “Hong Kong” pandemic (H3N2, 1968). H1N1 reemerged in 1977 and continued to circulate in the human population until 2009, when the pandemic H1N1 strain emerged. The continuity of the NP lineage along H1N1-H2N2-H3N2 viruses and the R-to-K changes are shown.

First, the amino acid sequences of A/Brevig/Mission/1918 and the TRIM22-resistant (WSN, PR8, pH1N1 [i.e., A/Paris/2590/2009]) and TRIM22-susceptible (A/USSR/90/77, sH1N1 [i.e., A/New Caledonia/20/99], A/Brisbane/59/2007 [12], and A/Paris/1149/2008) strains were aligned in chronological order. The overall homology of these sequences was >90%. As the lysine residues are potential sites for TRIM22-associated E3 ubiquitin ligase activity, we determined their numbers and positions. As shown in Fig. 3A, 15 lysine residues were conserved among TRIM22-resistant and TRIM22-sensitive strains (highlighted in green). Notably, when the NP sequence of the A/Brevig/Mission/1918 isolate was used as a reference, four arginine-to-lysine (R-to-K) changes (i.e., R98K, R293K, R422K, and R446K [highlighted in blue]) were observed in all TRIM22-sensitive H1N1 strains.

In order to trace the evolution of these 4 amino acids, we then analyzed the NP sequences of 7,690 H1N1 viruses available in the Influenza Research Database (IRD). The phylogenetic analysis of the sequences, chosen as the first in which the R-to-K changes appeared, clearly showed that the H1N1 seasonal strains that circulated prior to 2009 formed a monophyletic group originating from the 1918 pandemic strain, whereas the isolates derived from the 2009 H1N1 pandemic strain formed a distinct branch (Fig. 3B), confirming previous phylogenetic analysis (14, 15). Furthermore, the 4 arginine residues in the NP (R98, R293, R422, and R446) that characterized the 1918 pandemic H1N1 strain were still present in the subsequent seasonal viruses until 1936, when the first R-to-K substitution at site 98 was detected in the seasonal H1N1 A/Henry/1936 isolate. In 1940, an additional R422K change was present in the A/Hickox/1/1940 virus. The acquisition of a third mutation (R293K) was detected in the A/Albany/4835/1948 and A/Hemsbury/1948 viruses (not shown in the tree). H1N1 strains harboring K98, K293, K422, and R446 residues persistently circulated until 1957. The H2N2 and H3N2 reassortant viruses, which emerged in the human population in 1957 and 1968, respectively, and thereafter became predominant, retained the NP segment derived from the previously circulating H1N1 strains (16) and therefore showed the K98, K293, K422, and R446 residues in their NP. A virus of the H1N1 subtype (A/USSR/90/77) was reintroduced in 1977 (Fig. 3C). This virus was characterized by an additional R-to-K mutation at site 446, also present in two other H1N1 viruses circulating in the same year: i.e., A/Hong Kong/117/77 and A/Tientsin/78/77 (not shown in the tree). The K98-K293-K422-K446 NP signature was maintained in the seasonal H1N1 strains until 2009 (Fig. 3B, red asterisks), when the new 2009 pandemic H1N1 emerged. The 2009 pandemic NP sequence was characterized by four arginine residues at sites 98, 293, 422, and 446, similar to the NP sequence of the 1918 pandemic IAV. These four arginine residues have been retained so far in strains of the H1N1 pdm09 subtype (black asterisks in Fig. 3B). Furthermore, these four arginine residues are conserved in both avian and swine IAVs from the classical swine lineage, which was established in domestic pigs around 1918 and to which the NP segment from the 2009 pandemic H1N1 virus is phylogenetically related (16, 17).

Altogether, these findings strongly suggest that four R-to-K substitutions in NP occurred as a result of the sustained circulation of viruses derived from the 1918 IAV strain in the human population. We also analyzed the R-to-K mutations of 850 H3N2 NP sequences from 1968 to the present (last accessed 24 August 2017). As the NP segment of H3N2 viruses was acquired from the previously circulating H1N1 viruses upon genetic reassortment, the H3N2 NP of the 1968 strains is characterized by three lysine residues at sites 98, 293, and 422 and an arginine at site 446. The only amino acid that has changed since 1968 is at site 98. An arginine residue has been fixed at this position since 2004. Notably, in our previous study, we found that the A/Wisconsin/67/2005 H3N2 virus and the NYMC-X181 reassortant whose NP segment derives from the A/New York/55/2004 (H3N2) virus are susceptible to TRIM22 inhibition (12).

### Modeling of arginine-to-lysine NP adaptive mutations.

**FIG 4:**
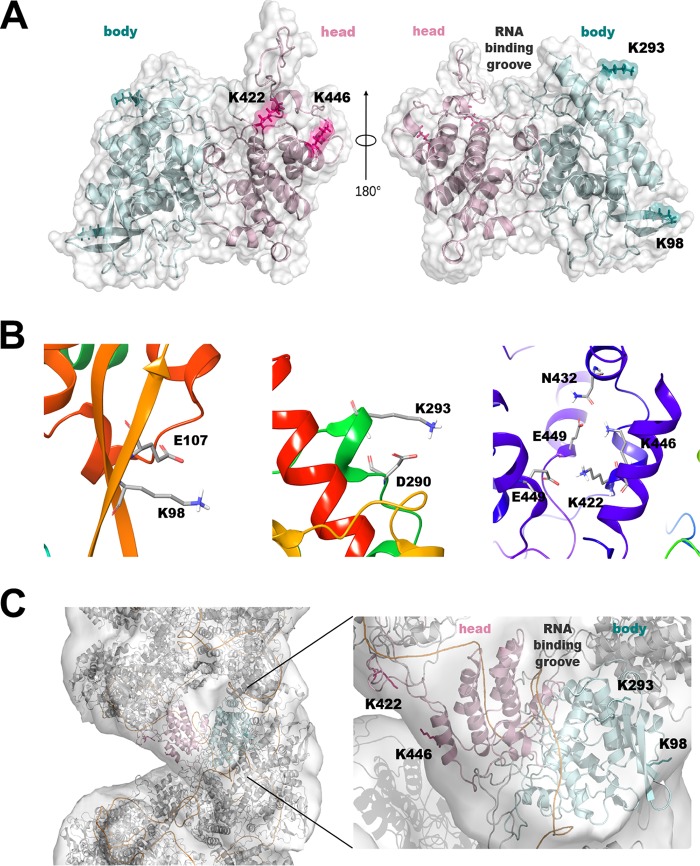
Localization of TRIM22-sensitive lysine residues in the NP molecular structure. Shown is a structural model of the mutant form of NP resistant to TRIM22 restriction. (A) The model of NPR4/K4, an NP with 4 R-to-K single-point mutations (R98K, R293K, R422K, and R446K), was generated using PDB code 2IQH as the template (18). NP is represented as cartoons, with the head and the body of the protein colored in pink and cyan, respectively. K98, K293, K422, and K446 are highlighted as sticks and numbered with the single-letter code. (B) The interactions predicted for K98 (left), K293 (middle), and K422 and K446 (right) are shown. Residues are highlighted as sticks and numbered with the single-letter code. (C) The model of NPR4/K4 in a helical ribonucleoprotein-like structure using PDB code 4BBL as the template (21) is shown as the surface. Images were prepared using pymol-v1.8.4.2 (PyMOL Molecular Graphics System, version 1.8; Schrödinger, LLC) and the Maestro software package (42).

We next examined the locations of amino acids 98, 293, 422, and 446 in the atomic NP crystal structure, which shows a noncontiguous main body and a head that are joined together by the polypeptide chain at three regions (18, 19). Based on the trimeric structure of NP from the A/WSN/33 H1N1 virus (PDB code 2IQH) (18), we generated *in silico* a corresponding mutant NP with R-to-K mutations at sites 98, 293, 422, and 446 (NPR4/K4), as described in Materials and Methods. The R-to-K mutations were not predicted to change the overall structure. Analysis of the interactions revealed that the four lysines create a similar, but not identical, interaction pattern compared to the corresponding arginines in the crystallographic structure. In detail, as shown in Fig. 4A, K98 and K293 reside on the main body, where they interact with the side chains of E107 and D290, respectively, (Fig. 4B, left and middle panels), whereas K422 and K446 are located on the head of the protein, where they contribute to homodimeric interactions (20). In particular, both K422 and K446 form a salt bridge with the corresponding E449 of a second NP molecule. Additionally, K446 forms a stabilizing electrostatic interaction with N432 (Fig. 4B, right panel). The major difference from the original NPR4 structure is that, unlike what was predicted for the K98 residue, the side chain of R98 interacts not only with the side chain of E107 but also with the side chain of E53 and the backbone of G54. Therefore, the K98 residue is predicted to weaken the network of electrostatic interactions observed in the crystallographic structure. We next calculated the solvent-accessible surface area (SASA) of NPR4/K4. All four lysines exhibited a relative side-chain SASA of >50%, similar to what was observed in the original NPR4 structure. Finally, to verify whether these four basic lysines could perturb RNA binding, we generated a complete model of NPR4/K4 in a helical ribonucleoprotein-like structure, as proposed by Arranz et al. (21) (PDB code 4BBL). According to this model, none of the lysines was located in the canonical RNA binding groove or interacted with RNA, no more than the four arginine residues in the NPR4 structure (Fig. 4C).

Overall, our modeling suggests that (i) the R-to-K mutations at positions 98, 293, 422, and 446 do not substantially modify the 3D structure of NP, and (ii) the four resulting lysines are exposed to the solvent and are therefore potential targets for ubiquitination.

### NP mutations and TRIM22 restriction of the viral polymerase activity.

**FIG 5:**
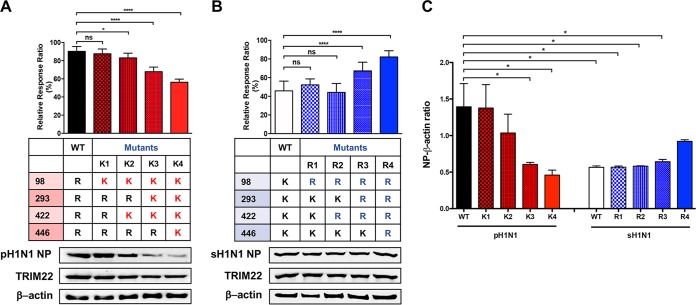
Arginine-to-lysine mutations conferring TRIM22 restriction and lysine-to-arginine mutations causing loss of TRIM22 restriction. TRIM22-mediated restriction activity was tested in the polymerase activity assay in the presence of 40 ng of TRIM22-expressing plasmid. (A) The bars show the activity of WT pH1N1 NP and R-to-K mutants relative to the same samples tested in the absence of TRIM22-expressing plasmid, which was set to 100%. Means ± SD from three independent experiments performed in triplicate are reported. The results of one-way ANOVA with the Bonferroni’s multiple-comparison test of WT versus each mutant are shown (*, *P* < 0.05; ****, *P* < 0.0001; ns, not significant). The expression levels of pH1N1 NP and TRIM22 were determined by Western blotting of WCE obtained by pooling the triplicate wells of each experiment. One representative Western blot of three is shown. (B) The bars show the activity of sH1N1 NP WT and K-to-R mutants relative to the same samples tested in the absence of TRIM22-expressing plasmid, which was set to 100%. Means ± SD from three independent experiments performed in triplicate are reported. The results of one-way ANOVA with the Bonferroni’s multiple-comparison test of WT versus each mutant are shown (****, *P* < 0.0001). Western blot analysis was performed to determine the expression levels of sH1N1 NP and TRIM22. (C) Quantification of the band intensity was performed by using ImageJ. Means ± SEM of the ratio between the intensity of NP bands and β-actin are reported for 3 independent experiments. The results of one-way ANOVA with the Bonferroni’s multiple-comparison test are shown (*, *P* < 0.05).

We next evaluated whether the four arginine residues identified by the *in silico* analysis were indeed necessary and sufficient to confer TRIM22 resistance. To this end, we generated R-to-K single, double, triple, and quadruple mutants in the pH1N1 NP backbone, thus mimicking the sequential changes that occurred during NP evolution from 1936 to 1977 (Fig. 3). All NP mutants were tested in the cell-based polymerase activity assay in the presence of TRIM22-expressing plasmid (40 ng), as described in the legend to Fig. 2. Although the R98K single mutation was not sufficient to confer TRIM22 restriction, the acquisition of two lysines (K98 and K422) rendered NP modestly but significantly susceptible to TRIM22 restriction. When three (K98, K293, and K422) or all four (K98, K293, K422, and K446) mutations were present, the susceptibility of polymerase activity to TRIM22 restriction progressively increased (Fig. 5A, upper panel).

In order to compare the steady-state levels of wild-type (WT) and mutant NP proteins in the presence of TRIM22, Western blot analysis was performed with whole-cell extract (WCE) obtained by pooling the triplicates from each experimental condition. As shown in Fig. 5A, lower panel, a representative Western blot analysis out of three revealed a progressive downregulation of NP from the K2 (K98 and K422) to K4 (K98, K293, K422, and K446) mutants. To further validate the importance of these lysine residues, we considered an opposite approach that consisted of mutating the lysine residues of sH1N1 NP backbone (i.e., NP from the A/New Caledonia/20/99 strain) into arginine residues to obtain the R1 to R4 mutants (Fig. 5B). Three K-to-R changes (mutant R3) were required to significantly decrease the sensitivity of the polymerase activity to TRIM22 restriction (Fig. 5B, upper panel). The Western blot analysis indicated that the expression of NP mutants was similar to that of WT NP (Fig. 5B, lower panel). In order to obtain quantitative data, the intensities of NP and of β-actin control bands were calculated as an NP/β-actin ratio. Consistent with TRIM22 inhibition of the polymerase activity, a significantly lower ratio was observed with WT sH1N1 NP versus WT pH1N1 NP (Fig. 5C). A significant progressive decrease of NP expression was present from K1 to K4 mutants, whereas the ratios of R1 to R3 mutants were similar to that of WT sH1N1 NP, with the exception of R4, in which expression showed a modest, although not statistically significant, increase. These results clearly show that the four lysine substitutions that have progressively occurred over time since 1918 confer progressive susceptibility to TRIM22, at least in terms of restriction of the polymerase activity.

### Arginine-to-lysine NP mutations and TRIM22-mediated NP ubiquitination.

**FIG 6:**
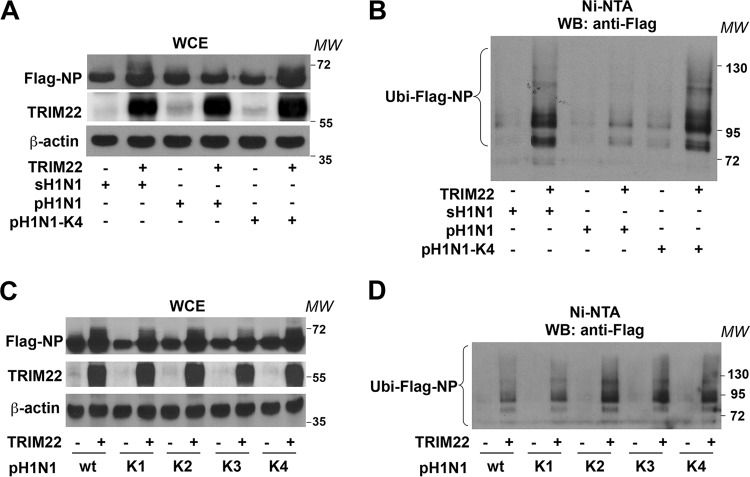
pH1N1 NP is less sensitive than sH1N1 NP to TRIM22-mediated ubiquitination. (A) sH1N1 Flag-NP, pH1N1 Flag-NP, and quadruple mutant (pH1N1-K4) were cotransfected with His-Ubi under all conditions and TRIM22 in HEK293T cells. WCEs were prepared from a 10% fraction of the cells and analyzed by Western blotting using anti-Flag (upper panel), anti-TRIM22 (middle panel), and anti-β-actin (lower panel) antibodies. (B) The remaining 90% of the cells were lysed under denaturing conditions, and ubiquitinated proteins were precipitated using Ni-NTA agarose. Purified ubiquitinated proteins were analyzed by Western blotting for the presence of ubiquitinated forms of Flag-NP (Ubi-NP) using anti-Flag antibodies. (C) The Flag-NP pH1N1 WT and its different K1, K2, K3, and K4 mutants were coexpressed with His-Ubi and TRIM22, in HEK293T cells. WCEs were prepared and analyzed as described for panel A. (D) Ubiquitinated forms of Flag-NP and its mutants (Ubi-NP) were analyzed as described for panel B. MW, molecular weight in thousands.

As we previously showed that the NP of seasonal strains is a target of TRIM22 E3 ubiquitin ligase activity (12), we tested whether the NP of pandemic strains could also be ubiquitinated by TRIM22. To this end, His-ubiquitin (His-Ubi) was cotransfected in HEK293T cells with Flag-NP and TRIM22 expression vectors in equal ratios. After 48 h, cells were incubated with the proteasome inhibitor MG-132 (10 µM for 3 h) in order to ensure accumulation of polyubiquitinated forms of NP. Ten percent of the cells were collected, and WCEs were analyzed by Western blotting using anti-Flag or anti-TRIM22 antibody (Ab). In the absence of TRIM22 expression, Flag-NP showed as a predominant band of ca. 62 kDa (Fig. 6A). Subsequently, the remaining 90% of the cells were lysed in a denaturing buffer containing 6 M guanidine-hydrochloride to dissociate potential ubiquitinated proteins bound to Flag-NP. Then, the ubiquitinated proteins were purified using Ni-nitrilotriacetic acid (NTA) beads and analyzed by 10% SDS-PAGE to resolve large-molecular-mass ubiquitin-conjugated NP products. Additional bands with a molecular mass larger than 62 kDa appeared when TRIM22 was coexpressed with ubiquitin, suggesting that TRIM22 induces mono- and polyubiquitination of NP; the ubiquitination activity of TRIM22 was less efficient with pH1N1 NP than sH1N1 NP (Fig. 6B). In contrast, with the quadruple pH1N1-K4 NP mutant (K98, K293, K422, and K446), TRIM22 expression induced levels of NP mono- and polyubiquitination similar to those obtained with sH1N1 NP.

We further investigated the TRIM22 ubiquitination activity against single (K98), double (K98, K422) and triple (K98, K293, and K422) pH1N1 NP mutants. Again as expected, in the absence of TRIM22 expression, Flag-NP showed as a predominant band of ca. 62 kDa (Fig. 6C), whereas additional bands with a molecular mass larger than 62 kDa appeared when TRIM22 was coexpressed with ubiquitin (Fig. 6D). The levels of TRIM22-mediated mono- and polyubiquitination were similar for the single (K1: K98) NP mutant and NP WT; the level was higher for the NP with two lysine substitutions (K2: K98 and K422) and did not increase further with three (K3: K98, K293, and K422) or four (K4: K98, K293, K422, and K446) lysine substitutions, suggesting that two lysine residues are sufficient to increase the efficiency of TRIM22-mediated ubiquitination of NP, at least in our transient-transfection assay.

These observations support the notion that TRIM22-mediated ubiquitination of NP could contribute, at least partially, to the progressive susceptibility to TRIM22 conferred by the sequential lysine substitutions.

### Lysine-to-arginine NP mutations and TRIM22 restriction of virus replication.

**FIG 7:**
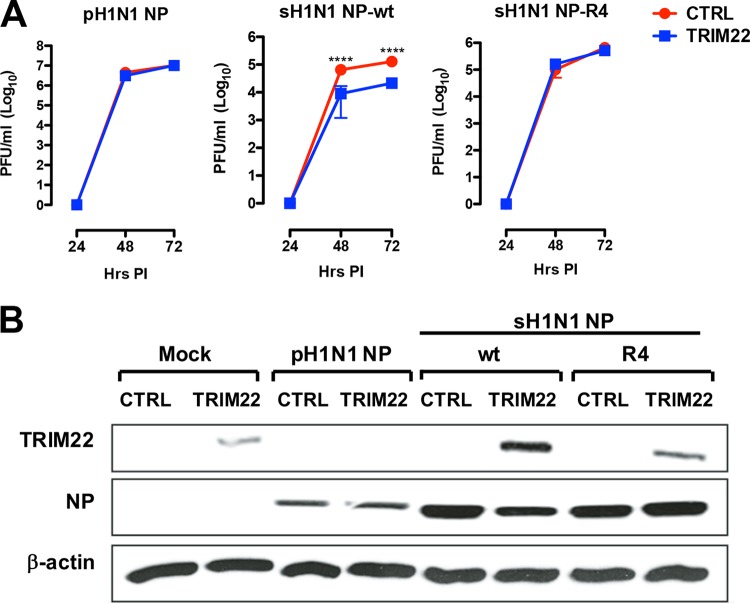
Four arginine-to-lysine residues render the sH1N1 NP sensitive to TRIM22 restriction in a viral growth assay. (A) Recombinant viruses were generated starting from a pandemic H1N1 system. These viruses were used to infect control and human TRIM22-transduced MDCK cells at an MOI of 0.001. Substitution of pH1N1 NP with sH1N1 NP WT increased the susceptibility to TRIM22 restriction activity, whereas mutant viruses generated through the introduction of the mutation cluster composed of 4 K-to-R changes (mutant R4) into sH1N1 resulted in a gain of TRIM22 resistance. Viral titers are expressed as the means ± SD from three experiments performed in duplicate. *P* values were determined using two-way ANOVA (****, *P* < 0.0001). (B) WCEs from control and TRIM22-overexpressing MDCK cells infected with the reverse-genetics viruses were analyzed by Western blotting assay, and viral NP expression levels were evaluated. TRIM22 expression was also examined, and β-actin was used as a normalizer. These results are representative of one out of three independent experiments.

In order to validate our findings in an infectious context, we produced a recombinant pH1N1 virus and two reassortant viruses harboring the NP segment from sH1N1—either WT or with four K-to-R substitutions at positions 98, 293, 422, and 446—and the remaining segments from pH1N1 by using the reverse-genetics system. The infectious titer of the two reassortant viruses was 1 log_10_ lower than those of the pH1N1 virus (Fig. 7A, compare the middle and right panels with the left panel). Interestingly enough, the pH1N1 recombinant virus replicated at similar titers in either the presence or absence of TRIM22 expression, whereas the replication of the reassortant pH1N1:sH1N1 virus was significantly reduced in the presence of TRIM22 (Fig. 7A). In contrast, the mutant reassortant virus (R4) grew to approximately the same infectious titers in either the presence or absence of TRIM22 (Fig. 7A). A Western blot analysis carried out at 72 hpi indicated that the NP accumulation levels were reduced in the presence of TRIM22 for the pH1N1:sH1N1 virus but not for the R4 mutant or the pH1N1 virus (Fig. 7B). It is interesting that TRIM22 expression was downregulated upon infection with the pH1N1 virus, but not with the reassortant viruses expressing the WT or R4 sH1N1 NP (Fig. 7B).

## DISCUSSION

We report here that the 2009 pandemic H1N1 virus as well as the WSN and PR8 strains, derived from the 1918 pandemic virus, are resistant to TRIM22 restriction, in contrast to what was observed for seasonal H1N1 strains that are inhibited by TRIM22. As TRIM22 targets the viral NP for polyubiquitination (12), we analyzed the numbers and sites of NP lysine residues in a large number of viral isolates of the IRD. We have identified four lysine residues (i.e., K98, K293, K422, and K446) that are potentially associated with sensitivity of seasonal IAV to TRIM22 restriction; in contrast, the pandemic strains are characterized by four arginine residues at the same sites in NP. Our data suggest that the progressive substitution of these lysine residues during prolonged IAV circulation in the human population is associated with increased sensitivity to TRIM22 restriction that is mediated by TRIM22 polyubiquitination and degradation of NP.

Successful establishment of pandemic IAV occurs when the newly introduced virus is efficiently transmitted from infected humans to humans immunologically naive (2). The selective advantage conferred by the new antigenicity often leads to the replacement of the formerly circulating seasonal IAV (22). Although innate and adaptive immune responses reduce serious illness and constrain the virus to progressively acquire features of annual seasonal strains (23), IAVs continuously evolve under natural and selective pressure of the host immune response (1). The IAV proteins that mostly accumulate selective mutations are the surface glycoproteins hemagglutinin (HA) and neuraminidase (NA), which are the main targets of the humoral response (24, 25). In addition, IAV internal genes are also under selective pressure of the adaptive immune response. In this regard, NP is a highly conserved (≈90%) internal IAV protein that nevertheless shows adaptive amino acid changes (16, 26). Indeed, most of the IAV immunodominant cytotoxic T lymphocyte (CTL) epitopes are located in NP (27); conversely, several amino acid substitutions of NP have been reported to correlate with the viral escape from the CTL response (28). Furthermore, comparison of CTL epitopes in human versus swine NP clearly indicates that human IAVs evolve under positive selection (29). Notably, two of the four NP residues that determine the sensitivity to TRIM22 (residues 98 and 422) are part of CTL epitopes, suggesting that the R98K and R422K mutations, which occurred earlier than R293K and R446K, may have been driven by the evasion of T-cell-mediated immunity. However, as residues 293 and 446 are not part of CTL epitopes, we cannot exclude other hypotheses, such as a positive selection of R-to-K substitutions driven by selection factors that are not related to T-cell-mediated immunity (e.g., increased sensitivity to TRIM22, by contributing to the attenuation of the virus, could indirectly favor viral spread) and/or a stochastic selection based on successive population bottlenecks.

The recombinant virus expressing sH1N1 NP lost TRIM22 restriction when all four lysine residues were replaced by arginine residues (R4 mutant), whereas three lysine-to-arginine changes at sites 98, 293, and 422 did not cause loss of TRIM22 restriction (data not shown), in contrast with the results obtained in the polymerase activity assay, (Fig. 5B), where the same substitutions caused loss of TRIM22 restriction. As four mutations are necessary to confer TRIM22 sensitivity in the viral growth assay, a partial discrepancy emerges by comparing it with the polymerase activity assay, in which only two mutations are enough to highlight an effect of TRIM22. These differences are likely explained by an intrinsic limitation of the polymerase activity assay to study the entire IAV life cycle (30, 31).

Notably, all four NP lysines required for TRIM22 restriction are exposed to the solvent and potentially accessible to its E3 ubiquitin ligase activity. Our previous work (12) and the present study have identified TRIM22 as an E3 ubiquitin ligase that promotes polyubiquitination of NP, therefore, causing its downregulation and inhibition of viral replication (Fig. 7). In addition, a recent independent report has shown that NP is endowed with multiple lysine residues that are sites of monoubiquitination by the CNOT4 (Ccr4-Not transcription complex subunit 4) protein (32). The same investigators have reported that NP is also a target of deubiquitinating enzymes such as USP11 (33). According to these studies, NP monoubiquitination plays a positive role in NP stability and overall polymerase function (33, 34). In this regard, it should be emphasized that the four lysine residues sensitive to TRIM22 polyubiquitination may not be a target of CNOT4 activity; however, it should be taken into account that CNOT4 activity was studied in the A/WSN/33 strain, which is insensitive to TRIM22 restriction and is characterized by four arginine residues at sites 93, 293, 422, and 446 (32). Nevertheless, both our and these independent results highlight the fact that IAV exploits the cellular ubiquitination machinery to either promote or limit viral replication. Of note, none of the amino acid variations observed in our study are involved in the conserved functional regions of NP, including the unconventional nuclear localization signal (NLS [residues 4 to 14]) and the bipartite NLS (residues 198 to 216) (35), binding to viral RNA (residues 73 to 91) (18) and viral polymerase (R204, W207, and R208) (36).

In contrast to TRIM22, MxA has been shown to be effective against avian strains, but not against seasonal viruses (9, 10). Interestingly, signatures of MxA resistance are present in NP of the 2009 pandemic H1N1 strain (10, 37). However, MxA escape mutations, which are a hallmark of all IAVs circulating in humans (10), initially caused a significant loss of viral fitness and were compensated for by the subsequent acquisition of additional mutations (37). Deep mutational scanning in MDCK cells expressing MxA has recently highlighted additional mutations that confer some level of MxA resistance and are conserved across human and avian influenza viruses (38). In order to explain these results, the authors have proposed that homologs of MxA in other hosts have selected some level of generalized MxA resistance in all NPs (38). However, an important difference between TRIM22 and MxA is the lack of *TRIM22* orthologs in avian and swine hosts (39). In addition to intrinsic innate immune differences between swine and human hosts, a recent analysis of NP evolution of the swine viral lineage has highlighted that swine influenza virus is not under the selection of CD8^+^ T cells and immune memory as pigs are infected only once or a few times during their short lives (29). Of note is the conservation of these four arginine residues (R98, R293, R422, and R446) in avian and swine NP (16, 17), supporting the importance of arginine-to lysine substitutions during IAV adaptation in the human host to endow the virus with TRIM22 restriction.

In conclusion, we have discovered that four arginine residues that were present in NP of seasonal H1N1 viruses in the 1930s evolved with time into lysines, leading to the acquisition of TRIM22-dependent inhibition of virus replication. Thus, this analysis has contributed to improving our understanding of NP genotype-to-phenotype mapping and in highlighting the complexity of the selective pressures acting on the NP, as adaptive changes inside but also outside CTL epitopes can be favored at the expense of increased sensitivity to some factors of the innate immune response to IAV infection.

## MATERIALS AND METHODS

### Cells and viruses.

HEK293T cells and Madin-Darby canine kidney (MDCK) cells were maintained in Dulbecco’s modified Eagle’s medium (DMEM) supplemented with glutamine (2 mmol/liter), penicillin (100 U/ml), streptomycin (100 U/ml), and 10% fetal bovine serum (FBS) (complete DMEM; Thermo Fisher Scientific, Inc.).

TRIM22-overexpressing MDCK cells were kindly provided by G. Towers (UCL, London, United Kingdom). Briefly, cells were transduced twice with a retroviral vector expressing TRIM22 (pEXN-HA-TRIM22) at 24-h intervals, replacing culture medium with vector-containing supernatant at a 1:1 ratio. MDCK control cells were transduced with an empty vector (pEXN-HA-empty). Seventy-two hours after the second transduction, cells were subjected to selection with G418 (3 mg/ml; Sigma-Aldrich).

Influenza virus strains A/WSN/33 (H1N1), A/PR/8/34 (H1N1), and A/Paris/7608/2009 (H1N1) were obtained by a reverse-genetics system. Other viruses were provided by the National Reference Center for Influenza Viruses at Institut Pasteur, Paris, France.

### IAV propagation and titration.

Monolayers of MDCK cells were washed twice with DMEM without serum and infected with H1N1 viruses at a multiplicity of infection (MOI) of 0.001. After virus adsorption for 1 h at 35°C, cells were washed twice and incubated at 35°C with DMEM without serum supplemented with tosylsulfonyl phenylalanyl chloromethyl ketone (TPCK)-treated trypsin (1 µg/ml; Worthington Biomedical Corporation). Supernatants were recovered 48 hpi.

For viral titration, a plaque assay was performed as previously described (12). Briefly, MDCK monolayer cells, seeded in 6-well plates at 1.2 × 10^6^/well, were washed twice with DMEM without serum, and serial dilutions of virus were adsorbed onto cells for 1 h. Cells were covered with MEM 2×-Avicel (FMC Biopolymer) mixture supplemented with TPCK-treated trypsin (1 µg/ml). Crystal violet staining was performed 48 hpi, and visible plaques were counted.

### Polymerase activity assay.

HEK293T cells (3 × 10^4^/well) were seeded into a 96-well plate. After 24 h, cells were transfected with a mixture of 4 plasmids encoding the components of the viral RNA polymerase complex (PB1, PB2, and PA [20 ng/each] plus NP [40 ng]) of the 2009 pandemic H1N1 strain under the promoter of the 3-hydroxy-3-methylglutaryl (HMG) coenzyme A reductase constitutively expressed in all cell types (40). In addition, plasmid pPR7-FluA-*Renilla* (5 ng), which encodes the viral minigenome, and a control plasmid expressing the firefly luciferase driven by the cytomegalovirus (CMV) promoter (5 ng) were included in the transfection mixture. To evaluate TRIM22 interference on virus-dependent transcription, HEK293T cells were cotransfected with increasing amounts of TRIM22-expressing plasmid. Cell lysates were harvested 48 h posttransfection. *Renilla* and firefly luciferase activities were determined using the Dual-Glo luciferase assay (Promega) according to the manufacturer’s instructions. Untransfected control cells were used to obtain the basal background.

### *In silico* analysis.

NP sequences of different human IAV H1N1 strains were analyzed using the IRD (http://www.fludb.org) of the U.S. National Institute of Allergy and Infectious Diseases through the Bioinformatics Resource Centers Program (last accessed 13 November 2016). NP sequences of each viral subtype were aligned using the MUltiple Sequence Comparison by Log-Expectation (MUSCLE) algorithm. The phylogenetic analyses were conducted with Molecular Evolutionary Genetics Analysis (MEGA; http://www.megasoftware.net). Bootstrap analysis was performed with 1,000 replications.

### Molecular modeling.

The three-dimensional (3D) model of the mutant form of NP, with 4 R-to-K single-point mutations at sites 98, 293, 422, and 446 (NPR4/K4), was generated using as the templates the crystallographic structure of the influenza A virus NP (PDB codes 2IQH [18] and 4BBL [21]). Models and missing loops were generated using the Swiss-model web server (https://swissmodel.expasy.org/) (41). Structures were then refined with the Protein Preparation Wizard available in the Maestro software package (42). Hydrogens were added, the orientations of the hydroxyl groups of serine, threonine, and tyrosine were obtained, and the side chains of asparagine and glutamine residues were optimized. The protonation states were chosen according to the neutral pH, and a minimization employing an opls 2005 force field with a root mean square deviation (RMSD) tolerance on heavy atom of 0.3 Å was performed. To evaluate the accessibilities of the four lysines (K98, K293, K422, and K446), we calculated the solvent-accessible area using Naccess V2.1.1 (43).

### Generation of recombinant IAVs.

The pandemic A/Paris/2590/2009 H1N1 strain was used in this study. WT pandemic virus was generated by reverse genetics using 8 bidirectional plasmids as previously described (44). NP mutant virus was generated by reverse genetics using a 9-plasmid reverse-genetics system, consisting of 7 bidirectional plasmids from the WT pandemic system, one monodirectional sH1N1 NP pPolI plasmid, which is in a negative orientation, and the pcDNA of the pH1N1 NP plasmid to increase the efficiency of reverse genetics. WT and mutant sH1N1 NP Ppr7 plasmids were synthetized by GenScript. Viruses were generated by 1-day coculture of HEK293T and MDCK cells (seeded at 3 × 10^5^ and 4 × 10^5^ cells, respectively, in 6-well plates) transfected with the plasmid mixture (0.5 µg per plasmid) using Lipofectamine 2000 (Invitrogen) according to the manufacturer’s recommendations. After 3 days, cell supernatants were harvested and clarified by low-speed centrifugation (2 min at 2,600 × *g* for 2 min at 4°C) and used to inoculate MDCK cells for 48 h for the amplification of rescued viral stocks. Viral titer was determined on MDCK cells by plaque assay. The virus NP gene was sequenced to validate the presence of the mutations.

### Site-directed mutagenesis.

To insert site-specific mutations in NP, site-directed mutagenesis was performed by using the QuikChange site-directed mutagenesis kit (Agilent). The arginine residues at sites 98, 293, 422, and 446 of pH1N1 NP were mutagenized into lysine residues, whereas sH1N1 NP was mutagenized to change lysine into arginine residues at the same positions. PCR amplification was performed using specific primer pairs. To fulfill the mutagenesis requirements, the primer pairs were designed to contain the desired mutation with a melting temperature (*T*_*m*_) of ≥68°C. The primer pairs were annealed to NP pcDNA3.1(+) plasmid.

### Western blot.

Whole-cell extract (WCE) from MDCK and HEK293T cells was prepared as previously described (12). Samples were run on SDS-polyacrylamide gel electrophoresis (PAGE) and transferred to nitrocellulose membrane by electroblotting. Filters were incubated with either a rabbit polyclonal Ab raised against TRIM22 (45) or an anti-NP monoclonal antibody (MAb; Southern Biotech). An anti-β-actin MAb (Sigma-Aldrich) was used as a control. Quantification of bands was performed by using ImageJ software (version 1.47v, WS Rasband ImageJ, NIH; http://rsb.info.nih.gov).

### Ubiquitination assay.

WT and mutant pH1N1 NP coding sequences were cloned from pcDNA3.1(+) into p3×Flag-Myc-CMV-26 plasmid (Sigma-Aldrich). Transient-transfection experiments were performed in HEK293T cells in 100-mm cell culture dishes using the calcium phosphate method (2 µg of Flag-NP plus 4 µg of His-Ubi plus 4 µg of TRIM22). Forty-eight hours after transfections, cells were treated with 10 µM MG132 for 3 h. Cells were washed twice in phosphate-buffered-saline (PBS) and resuspended in lysis buffer (20 mM Tris-HCl [pH 8], 10 mM NaF, 1 mM EDTA, 150 mM NaCl, 1 mM dithiothreitol [DTT], 100 µM phenylmethylsulfonyl fluoride [PMSF], phosphatase, and protease inhibitor cocktail [1 tablet/10 ml; Roche Diagnostics], 10% glycerol, 1% NP-40). For Ni-NTA (Qiagen) pulldown experiments, WCE was prepared from a 10% fraction of the cells and analyzed by Western blotting for the expression of transfected proteins. The remaining 90% of the cells were lysed in a denaturing buffer (6 M guanidine-HCl, 100 mM Na_2_HPO_4_/NaH_2_PO_4_ [pH 8.0], 10 mM Tris-HCl [pH 8.0], 0.2% Triton X-100), and ubiquitinated proteins were precipitated using Ni-NTA agarose.

### Statistical analysis.

All statistical analyses were performed using Prism GraphPad Software, Inc., v.4.0 (GraphPad Software, Inc.). Comparison between two groups was performed using the unpaired *t* test, while two-way analysis of variance (ANOVA) with Bonferroni’s multiple comparison was used to compare more than two groups.
